# Spontaneous Epidural Hematoma in Patients With Sickle Cell Disease: A Series of Two Cases of a Rare Neurological Complication

**DOI:** 10.7759/cureus.97596

**Published:** 2025-11-23

**Authors:** Vimukta Pradhan, Himanshu Shekhar, Abhishek Kumar Sinha, Punam Kumari Munda

**Affiliations:** 1 General Medicine, Mahatma Gandhi Memorial Medical College and Hospital, Jamshedpur, IND; 2 General Medicine, Medinirai Medical College, Palamu, IND; 3 General Medicine, Rajendra Institute of Medical Sciences, Ranchi, IND; 4 Ophthalmology, Shaheed Nirmal Mahato Medical College, Dhanbad, IND

**Keywords:** epidural hematoma, mr venography, neurological complication, sickle cell crisis, sickle cell disease

## Abstract

An epidural hematoma (EDH) arises most commonly in cases of cranial trauma. Sickle cell disease (SCD) is a hemoglobinopathy associated with multiple complications, and spontaneous EDH is one of its rare complications that usually arises during the crisis phase. The proposed mechanisms include skull bone infarction, cortical disruption, and marrow expansion, which can lead to hemorrhage. Owing to its rarity, it continues to pose diagnostic challenges for clinicians. We report two cases of spontaneous EDH with distinct clinical presentations and management. The first case was of an 18-year-old homozygous SCD patient, who presented with headache and nonspecific symptoms during a crisis. Neuroimaging showed an EDH, along with a subgaleal hematoma. The patient was managed conservatively with good clinical outcomes. The second case was of a young female who developed a rapidly progressive neurological deficit, and imaging showed an extensive epidural hematoma requiring urgent surgical decompression. The patient improved subsequently. These cases emphasize the significance of a high index of suspicion for EDH during the crisis phase of SCD for timely management and better clinical outcomes.

## Introduction

An epidural hematoma (EDH) is a potentially life-threatening neurological emergency characterized by the accumulation of blood between the dura mater and the inner table of the skull and is seen frequently after cranial trauma. Spontaneous EDH arises from non-traumatic etiologies such as vascular malformations, infections, or hematologic disorders [1].

Sickle cell disease (SCD) is an inherited hemoglobinopathy characterized by the production of abnormal hemoglobin, which results in deformed, sickle-shaped red blood cells that lead to small blood vessel occlusion, causing repeated episodes of ischemia, chronic hemolysis, and a wide range of systemic complications [2].

Among the various systems involved, complications of the nervous system are common in SCD crises. Neurologic complications are reported in 6-30% of patients with SCD [3,4]. Ischemic stroke is well known in SCD and occurs mostly in children [5]. Hemorrhagic events are far less common, about 3-10% of all strokes, but usually carry a poor prognosis [3,5]. Most documented cases involve bleeding into the subarachnoid or intracerebral spaces. Spontaneous EDH, on the other hand, constitutes only a minute fraction of the hemorrhagic spectrum described in contemporary series and reviews [6,7].

The rarity of this complication is evident by the limited number of cases reported in the literature. The most comprehensive scoping review to date, published by Patel et al. in 2025 [7], identified only 71 previously documented cases of spontaneous EDH in patients with SCD worldwide till mid-2024 and added three new cases from their own series, bringing the total number to 74 documented cases in that review. A subsequent literature search performed in November 2025 revealed only four recent additional cases in reports published since then, including those by Taylor et al. (2024) [8], Adegboye et al. (2025) [9], and Nsengiyumva et al. (2025) [10]. This brings the cumulative number of documented cases to less than 80 worldwide. Although, this figure may not represent the absolute total number of global cases, due to publication delays and limited indexing of regional journals, this exceptionally small number underscores not only the rarity of this event but also the need for a thorough reporting of all cases, as detailed documentation is essential for developing a clearer understanding of its epidemiology.

During a vaso-occlusive crisis, an EDH occurs even in the absence of any trauma. Symptoms are often nonspecific and overlap with those of a vaso-occlusive crisis, leading to diagnostic delay. Early recognition and prompt intervention are crucial, given the potential for rapid neurological deterioration and adverse clinical outcomes [11]. The presentation may be subtle at first but can deteriorate rapidly.

This report describes two unusual cases of a spontaneous EDH in patients with underlying SCD, emphasizing the vivid clinical presentation, diagnostic challenges, possible underlying mechanisms, and different management strategies based on their individual clinical presentation.

A review of the available literature reveals that precise data on incidence and epidemiologic details for spontaneous EDH in patients with SCD is lacking. Consequently, current understanding of this entity remains limited, to a large extent, on case reports. In the absence of larger datasets, each additional well-structured case report provides meaningful insight for enhancing clinical recognition, improving management protocols, and gradually shaping a more evidence-based approach to prognosis and patient care. These two reports enrich the scarce literature, outlining the variable clinical and radiological presentations, possible underlying mechanisms, and differing management strategies of spontaneous EDH in patients with SCD, and highlights the importance of timely diagnosis and early intervention.

## Case presentation

Case 1

An 18-year-old boy, a known case of SCD with genotype HbSS, presented to a tertiary care center in vaso-occlusive crisis with a five-day history of fever and cough, along with severe body ache and a progressively worsening headache for two days. On examination, the patient was conscious with a Glasgow Coma Scale (GCS) score of 15/15, febrile, hemodynamically stable, and exhibited pallor. A boggy, fluctuant, non-tender scalp swelling with ill-defined margins was noted in the frontoparietal region bilaterally, without any history of trauma. There were no neurological deficits, and transillumination was negative.

**Figure 1:**
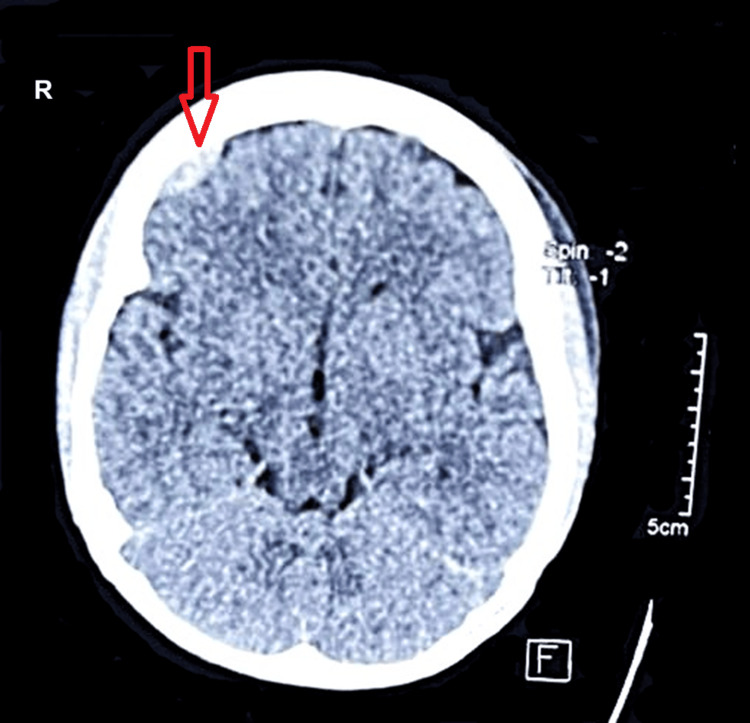
Axial non-contrast CT of the head showing the right frontal epidural hematoma (arrow) Non-contrast axial CT of the head showing a right frontal well-defined biconvex hyperdense collection of hemorrhagic attenuation of 45 HU, suggestive of epidural hematoma.

**Figure 2:**
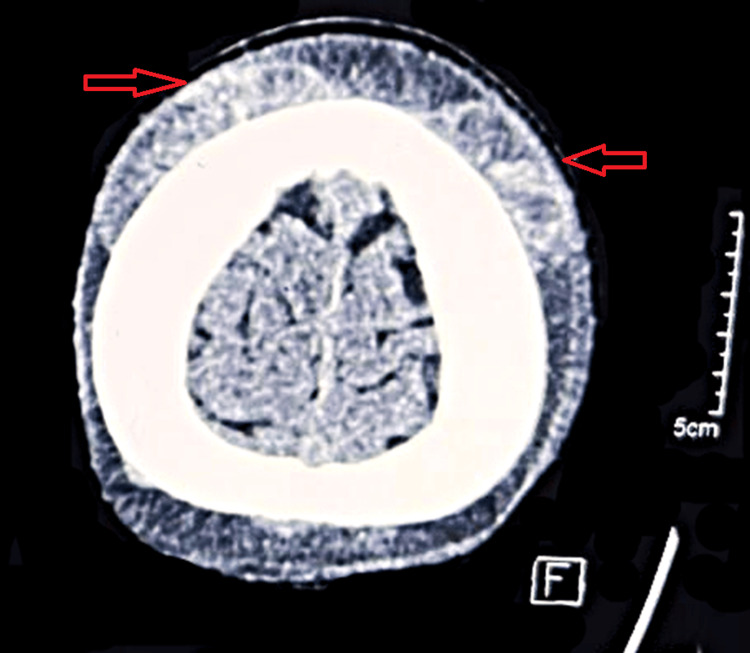
A non-contrast CT of the head showing the subgaleal hematoma (arrows) Non-contrast axial CT of the head showing the extracalvarial hyperdense region of hemorrhagic attenuation of 45 HU in subgaleal space of the bilateral frontoparietal region, suggestive of subgaleal hematoma.

Initial laboratory investigations revealed severe anemia (hemoglobin 6.9 g/dL; reference ranges: 13.0-17.0 g/dL (male) and 12.0-16.0 g/dL (female)), leukocytosis (WBC 16,650/μL; reference range: 4,000-11,000 /µL), and a normal coagulation profile (INR 0.97 (reference range: 0.86-1.14; activated partial thromboplastin time or aPTT 29 sec (reference range: 25-36 seconds)). Cerebrospinal fluid (CSF) examination was normal. Liver function tests showed mild transaminitis and indirect hyperbilirubinemia with total bilirubin at 2.6 g/dL (reference range: 0-1.2 mg/dL) and indirect bilirubin at 1.8 g/dL (reference range: 0-0.5 mg/dL). Serum lactate dehydrogenase (LDH) was elevated to 432 U/L (reference value: up to 250 U/L). Thrombotic markers (Protein C activity 80%, Protein S activity 78%, and Antithrombin III 0.25 g/L) were within normal limits, and autoimmune screening for anti-nuclear antibodies (ANA) was negative. A non-contrast CT (NCCT) scan of the head done on the day of admission revealed a small right high frontal well-defined biconvex hyperdense collection suggestive of EDH associated with a well-defined extra calvarial hyperdense region of hemorrhagic attenuation in the subgaleal space of the bilateral frontoparietal region, right more than left, and of maximum thickness 14 mm, suggestive of subgaleal hematoma (Figures 1, 2).

An MRI of the head was consistent with a right frontal EDH with bilateral frontoparietal subgaleal blood collections. The MRI also showed T1-hypointensity and T2-weighted short-tau inversion recovery (T2/STIR) hyperintensity in the calvarial marrow, consistent with bone marrow edema. Diffusion-weighted imaging (DWI) demonstrated a high DWI signal with corresponding low apparent diffusion coefficient (ADC) values, indicating diffusion restriction with focal areas of sclerosis suggestive of skull bone infarction. The patient was diagnosed with spontaneous EDH secondary to sickle cell crisis. He was managed conservatively with blood transfusion, broad-spectrum antibiotics, non-steroidal anti-inflammatory drugs (NSAIDs), oxygen, and supportive care. Neurosurgical intervention was not done, as the initial EDH was small in size, and repeat imaging after one week showed no progression or mass effect. After 10 days of hospital stay, the patient was discharged in a stable condition on hydroxyurea and folic acid tablets. He was advised to have regular neurological and hematological follow-up. Clinical reviews were conducted at four weeks and eight weeks after discharge. He remained completely asymptomatic, with no headache, vomiting, or neurological deficits on examination. Although a follow-up CT scan was strongly recommended to document radiological resolution of the epidural hematoma, the patient declined repeat imaging due to financial constraints and absence of symptoms. Clinical stability and recovery were therefore used as indicators of resolution.

Case 2

**Figure 3:**
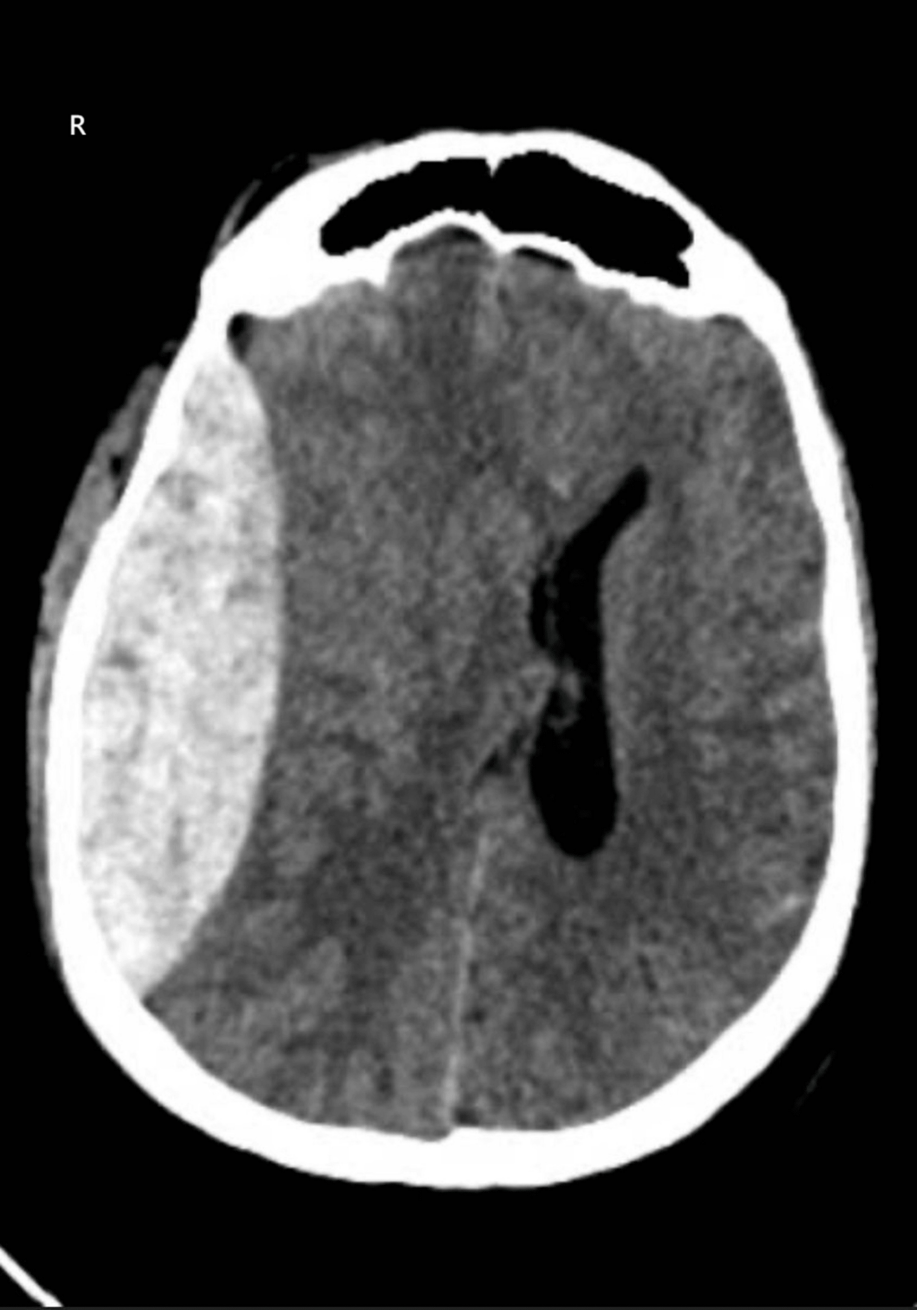
Non-contrast axial CT of the head showing right frontoparietal epidural hematoma Non-contrast axial CT of the head showing a large biconvex (lentiform), hyperdense collection along the right frontoparietal convexity in the extra-axial location, causing mass effect with midline shift, consistent with an acute epidural hematoma in the right frontoparietal region.

A 15-year-old female patient, a known case of SCD with genotype HbSS, presented with a three-day history of back pain, chest pain, generalized body aches, and bilateral thigh pain. She was diagnosed with sickle cell vaso-occlusive crisis and was being managed conservatively. On day four, the patient developed nausea, a severe headache, episodes of recurrent vomiting, and diplopia. There was no history of trauma. On examination, the patient was conscious, oriented, and irritable. Her vitals were stable, but neurological examination revealed focal deficits on the left side, with right lateral rectus palsy. Within hours of clinical examination, her GCS score, which was 13/15 initially, rapidly declined to 7/15, accompanied by an altered level of consciousness. Blood, urine, and CSF samples were collected for investigation. Laboratory investigations revealed anemia (hemoglobin 8.9 g/dL) with leukocytosis (WBC 14,400/μL) and evidence of hemolysis, indicated by mildly elevated serum lactate dehydrogenase (LDH; 320 U/L) and indirect bilirubin 1.3 mg/dL. CSF analysis was normal. Screening for ANA was negative. Coagulation studies, including aPTT (32 sec), Protein C (90% activity), Protein S (78% activity), and Antithrombin III (0.28 g/L), were normal. Serum homocysteine was 6.85 µmol/L and within normal limits. An NCCT scan of the head revealed a right frontoparietal well-defined biconvex hyperdensity suggestive of EDH with an approximate estimated volume of 180 cc, with midline shift and mass effect (Figure 3).

Based on the findings, the patient underwent emergency decompressive surgery with hematoma evacuation. Postoperatively, she regained consciousness and was subsequently extubated on day two after surgery. Episodes of vomiting subsided, neurological status improved steadily, diplopia resolved by day three, and left-side upper and lower limb power improved in the first week to medical research council (MRC) muscle strength score to 3/5. By day five, she was appropriately interactive and participating in active physiotherapy. By Day 10, she had regained functional mobility with only minimal residual weakness. She remained stable with continued supervised rehabilitation. Her postoperative NCCT brain, obtained after 72 hours, showed a postoperative craniotomy defect over the right frontoparietal region with complete evacuation of the previously noted epidural hematoma. The underlying brain re-expanded, midline structures were central, and basal cisterns were well maintained. No residual hematoma or new hemorrhage was noted. Small postoperative pneumocephalus was seen, which is expected. CT angiography showed normal intracranial arterial opacification with no aneurysm, arteriovenous malformation, dural arteriovenous fistula, vascular malformation, or abnormal vascular blush. A CT scan one day before discharge shows no residual hematoma, no new bleed, and a normal brain parenchyma. The patient was discharged in stable condition on day 14 with an outpatient physiotherapy plan. Despite being counseled regarding the need for regular follow-up and repeat imaging, the patient was lost to follow-up and denied further investigations and evaluation, citing financial constraints. Table 1 below shows a comparative analysis of both the cases.

**Table 1:** Comparative summary of the two reported cases Comparison of the key clinical features, initial presentation, diagnostic findings, management strategies, and postoperative outcomes of the two patients included in this case series. VOC: Vaso-occlusive crisis; WBC: White blood cells; LDH: Lactate dehydrogenase; AT-III: Antithrombin III; NCCT: non-contrast CT; EDH: epidural hematoma; aPTT: activated Partial Thromboplastin Time.

Parameter	Case 1	Case 2
Age/Sex	18-year-old, male	15-year-old, female
SCD genotype	HbSS	HbSS
Presenting complaints	VOC with headache, fever, boggy scalp swelling	VOC with body ache, which progressed to nausea, vomiting, diplopia, and rapid neurological deterioration
GCS score	15/15	8/15
Scalp findings	Subgaleal hematoma over the bilateral fronto-parietal region	No subgaleal hematoma
Lab findings	Anemia (Hb 6.9 g/dL), WBC 16,650/µL, LDH (432 U/L), indirect hyperbilirubinemia	Anemia (Hb 8.9 g/dL), WBC 14,400/µL, LDH elevation (320 U/L), indirect bilirubin 1.3 mg/dL
Coagulation profile	Normal (INR 0.97; aPTT 29 sec), Protein C/S/AT-III = Normal	Normal (INR 1.01; aPTT 32 sec) Protein C/S/AT-III = normal
NCCT brain	Small right high frontal EDH + bilateral frontoparietal subgaleal hematoma	Large right frontoparietal EDH (~180 cc) with midline shift
Management	Conservative	Neurosurgical decompression
Hospital stay	10 days	14 days
Outcome	Full recovery	Ambulatory with minimal weakness; stable

## Discussion

Among CNS complications in SCD, spontaneous EDH happens to be a rare entity [11]. Although precise data on its incidence are limited, spontaneous EDH in SCD is increasingly recognized in published case series [11]. India carries the second-highest burden of SCD globally [12]. The condition is heterogeneously distributed across the country and shows a higher prevalence among tribal populations [13]. Both our patients belonged to tribal communities from eastern India, which aligns with the well-known epidemiological trend. Both cases are in line with the literature review, demonstrating that spontaneous EDH is most often associated with the HbSS genotype [14]. Joy et al. in 2022 reported that spontaneous EDH occurs more frequently in males, and Patel et al. reported an 84% male preponderance. Interestingly, among the two cases, one is a male patient and the other is a female patient [15].

Elevated leukocyte counts are documented in cases of intracranial hemorrhage in SCD, including the patients reported herein. Nevertheless, the precise pathogenic role of leukocytosis in the evolution of EDH is not well established [11].

The exact pathophysiological mechanism remains unclear, but it is thought to involve an ischemic infarction of the skull bones, which leads to increased vascular fragility and hemorrhage, even in the absence of trauma [16]. Venous stasis and microvascular occlusion from sickled erythrocytes during crisis increase the risk of bone infarction [17]. A study by Saha and Saha (2019) highlighted that bone infarctions in SCD patients could lead to EDH [11]. A skull bone infarction causes an elevation of the periosteum, which injures the vessels and causes bleeding intracranially into the epidural space and sometimes also into the subgaleal space [11,16]. The coexistence of subgaleal hematoma with EDH in our first case suggests this pathophysiology.

The first case demonstrates imaging findings of bone marrow edema and sclerosis, which are consistent with acute bone infarction. Another postulated mechanism involves rapid hematopoiesis and expansion of the bone marrow with resultant microfracture of the already thinned inner cortex and extravasation of blood and hematopoietic tissue [17].

Additionally, the fragile diploic veins and thinning of the cortical bone in SCD patients further increase the likelihood of spontaneous bleeding. Dahdaleh et al. in 2009 have shown that the chronic disease process in SCD with increased medullary hematopoiesis changes the structure of the skull bones, leading to spontaneous subgaleal hematoma, as manifested in our first case [17]. However, in our second case, the exact pathophysiological mechanism of bleeding could not be delineated. The event can be unifactorial or multifactorial, with the interplay of several mechanisms acting together [11,17].

The clinical presentation of spontaneous EDH can be variable. It can range from nonspecific symptoms resembling sickle cell crisis, such as pain, fever, and headache, as in our first case, to obvious neurological symptoms and experiencing acute neurological deterioration, such as focal deficits and diplopia during a vaso-occlusive crisis, as in our second case [18]. Timely radiological scans, such as CT and MRI, are critical in diagnosing EDH and assessing its severity and thereby planning the appropriate intervention [19]. Across several case series and literature reviews, spontaneous EDH in SCD has been associated with a mortality rate of nearly 20% [7,15,20]. Some of the case reports in literature have good clinical outcomes on conservative management [21,22]. Management is usually conservative for small, non-compressive hematomas, as in our first case. However, urgent surgical decompression is warranted for larger hematomas producing a mass effect, as observed in our second case [16-19]. Although surgical intervention is not always required in spontaneous EDH, timely diagnosis is required for improved clinical outcomes [18,19]. A comparative overview of previously published cases and the present cases is shown in table 2.

**Table 2:** Comparative overview of the previously-published and present cases Comparison of the patient demographics, clinical presentation, imaging features, management strategies, and outcomes between published reports of spontaneous epidural hematoma (EDH) in sickle cell disease (SCD) and the present cases.

Study (Year)	Type of study	Age/Sex	Presentation	Imaging	Management	Outcome
Resar et al. (1996) [16]	Case report	14/M	Headache, vomiting	Skull infarction with right EDH	Surgical evacuation	Recovered
Dahdaleh et al. (2009) [17]	Case report	15/M	Headache, scalp swelling	Skull infarction with EDH and subgaleal	Conservative	Recovered
Sangle et al. (2011) [23]	Case report	15/M	Headache, vomiting	Spontaneous EDH	Not specified	Deceased
Hettige et al. (2015) [14]	Case report & literature review	18/M	Severe headache, focal deficit	Frontoparietal EDH	Surgical evacuation	68.2% Survival 18.2% Mortality
N’Dri Oka et al. (2015) [21]	Case report	12/F	Headache, fever, drowsiness	Right parietal EDH	Surgical evacuation	Recovered
Hamm et al. (2017) [20]	Case series	10-18/M	Variable	Spontaneous EDH	Surgical evacuation	22.2% Mortality
Saha and Saha (2019) [11]	Case report	19/M	Headache, scalp swelling	Bifrontal EDH with bone infarction	Conservative	Recovered
Iversen et al. (2019) [19]	Case report	16/M	Headache, vomiting, weakness	Massive extradural hematoma	Surgical Evacuation	Recovered
Kotey et al. (2020) [24]	Case report	18/M	Scalp swelling, headache, vomiting	Extensive EDH & corpus callosum bleed	Could not be operated	Deceased
Joy et al. (2022) [15]	Case report	20/M	Headache, vomiting, weakness	Frontal EDH	Surgical evacuation	Recovered
Present Case 1	Case report	18/M	Headache, scalp swelling	High frontal EDH	Conservative	Recovered
Present Case 2	Case report	15/F	Headache, nausea, vomiting, diplopia, weakness	Fronto-parietal EDH	Surgical evacuation	Recovered with minimal residual weakness

The possible differentials to consider in a case of non-traumatic spontaneous EDH include bleeding diathesis, vascular malformations of the dura mater, and metastasis to the dura or skull, all of which were ruled out with investigations. Coagulation disorders were ruled out in both the patients, with normal findings for aPTT and antithrombin III and activities of Protein C and Protein S level in the lower normal range. These findings support the likelihood of a sickle-cell-related spontaneous hemorrhage rather than a coagulopathy. The patient's CSF analysis was also normal, thereby helping to exclude infectious causes. The absence of vascular abnormalities on magnetic resonance venography suggested that the EDH was probably related to the sickling pathology, rather than an underlying arteriovenous malformation or aneurysm. This is consistent with findings by Iversen et al. (2019), which suggested that coagulopathy-related EDH is uncommon in SCD patients, with the primary cause of bleeding being related to bone infarctions rather than clotting disorders [19].

Although uncommon, spontaneous EDH in SCD can be life-threatening. Early suspicion, prompt imaging, and timely intervention are essential to prevent irreversible neurological damage. These two cases underscore the importance of considering EDH in patients with SCD, as early intervention can prevent permanent neurological deficits. Some cases resolve with conservative management and don’t require any surgical intervention unless there is a mass effect, midline shift, or abscess formation due to secondary infection of subgaleal hematoma.

Limitations

The main limitations of these two case reports include the failure to obtain long-term follow-up for either patient, limiting our ability to assess sustained clinical and radiological outcomes. In the second case, early loss to follow-up further restricted longitudinal evaluation, and the precise pathophysiological mechanism underlying the spontaneous EDH could not be definitively established. Additionally, the small sample size inherent to two case reports, limits the generalizability of our observations. Larger studies with extended follow-up are needed to better elucidate causation and clinical trajectories in such patients. Despite these limitations, the cases provided valuable insight into the variable presentation, diagnostic challenges, and management strategies for spontaneous EDH in SCD.

## Conclusions

These two case reports highlight an uncommon instance of spontaneous EDH in a patient with SCD, presenting as a complication of vaso-occlusive crisis, underscoring the importance of prompt imaging and early surgical intervention. Although rare, these complications can become life-threatening when large hematomas cause mass effect, warranting urgent neurosurgical intervention. Prompt intervention is lifesaving with excellent outcomes. A medical professional should always be vigilant for early signs of EDH in cases of sickle cell crisis presenting with acute neurological symptoms. In rare scenarios like these, early neuroimaging and timely intervention are paramount for reducing morbidity and mortality.
